# Gut colonization with vancomycin-resistant *Enterococcus* and risk for subsequent enteric infection

**DOI:** 10.1186/s13099-018-0259-4

**Published:** 2018-07-09

**Authors:** Jordan E. Axelrad, Benjamin Lebwohl, Edward Cuaresma, Ken Cadwell, Peter H. R. Green, Daniel E. Freedberg

**Affiliations:** 1 Division of Gastroenterology, Department of Medicine, Inflammatory Bowel Disease Center, NYU Langone Health, 240 East 38th Street, 23rd Floor, New York, NY USA; 2 Division of Digestive and Liver Disease, Department of Medicine, Columbia University Medical Center, New York, NY USA; 3 Kimmel Center for Biology and Medicine at the Skirball Institute, New York University School of Medicine, New York, NY USA; 4 Department of Microbiology, New York University School of Medicine, New York, NY USA

**Keywords:** Vancomycin-resistant enterococci (VRE), Multiplex PCR testing, Enteric infection, *Clostridium difficile*

## Abstract

**Background:**

Gut colonization with vancomycin-resistant *Enterococcus* (VRE) is associated with poor outcomes. This study evaluated the impact of VRE colonization on subsequent acquisition of enteric pathogens.

**Methods:**

We performed a retrospective cohort study of adults admitted to an ICU from 2012 to 2017 who were screened for VRE colonization and subsequently underwent stool testing with a gastrointestinal pathogen PCR panel (GI PCR) with or without PCR testing for *Clostridium difficile*. Our primary outcome was the presence of any enteric pathogen. Cox proportional hazards modeling was used to adjust for factors associated with enteric infection.

**Results:**

Of 761 patients who underwent VRE screening and subsequent GI PCR, 131 (17%) were colonized with VRE. Patients with VRE colonization were less likely to test positive on GI PCR compared to patients without VRE (9.2% vs 18%, p = 0.01); specifically for *E. coli* species (p = 0.03) and viral (p = 0.04) enteric infections. In 716 patients who underwent *C. difficile* testing, there was a trend towards more *C. difficile* infections in patients colonized with VRE (15% vs 10%, p = 0.11). On multivariable analysis, patients with VRE had a decreased risk of a positive GI PCR (aHR 0.47, 95% CI 0.25–0.88, p = 0.02) but not *C. difficile* infection, effects which persisted during 5 years of follow-up. Among positive tests, there was a greater proportion of *C. difficile* with VRE (57% vs 28%, p < 0.01).

**Conclusions:**

VRE colonization was associated with a decreased risk of subsequent non-*C. difficile* enteric infection. VRE domination of the gut microbiome may protect against acquisition of common enteric pathogens.

## Background

Vancomycin-resistant enterococci (VRE) have emerged as one of the most important nosocomial pathogens and may result in severe bloodstream, intra-abdominal, and urinary tract infections [1–3]. Typically, asymptomatic VRE gut colonization precedes infection with susceptible hosts comprising patients who are severely ill, exposed to multiple and prolonged courses of antimicrobial agents, hospitalized for long lengths of stay (LOS), residing in a long-term care facility, located in close proximity to another colonized or infected patient, or hospitalized in a room previously occupied by a patient colonized with VRE [1, 4, 5]. Colonization may persist for weeks to years [6–8]. Intensive Care Units (ICUs) are common reservoirs for antibiotic resistant organisms including VRE with rates of colonization via rectal swab ranging from 9.7 to 51.9% [9, 10].

The clinical implications of VRE infection include the limited availability of antimicrobial therapies against VRE and the ability of VRE to transfer the genetic determinant for vancomycin resistance to other pathogens [6, 11]. VRE colonization is also associated with worse clinical outcomes. In a recent propensity score matched cohort study comparing the outcomes of patients with VRE colonization to those without colonization at the time they were admitted to the ICU, VRE colonization was associated with increased mortality, LOS, and costs [9].

Enteric infection is a major cause of morbidity and mortality, and infection in compromised patients may result in more severe illness [12, 13]. In recent years, highly sensitive and specific molecular multiplex PCR assays have started to replace conventional microbiological tests as a rapid and accurate means of diagnosing enteric infection [14, 15]. Despite the recognition of VRE as a dangerous, health care-associated infection, little is known regarding the impact of gut VRE colonization on the acquisition of other enteric pathogens. When present in hospitalized patients, VRE often dominates the gut microbiome. In bone marrow transplant patients who are exposed to multiple antibiotics or ICU patients with prolonged stays, VRE frequently constitutes over 30% of all gut bacteria [16]. In these or similar at-risk patients, VRE appears to displace commensal anaerobes but whether it also displaces enteric pathogens has not previously been studied. The objectives of the present study were to evaluate the risk, risk factors, and pathogenic distribution of enteric infection in patients colonized with VRE using PCR-based stool tests.

## Results

Of 3330 patients screened for VRE on admission to an ICU, 371 (11%) were positive for VRE and 761 (23%) underwent subsequent stool PCR testing. Patients with VRE were more likely to undergo stool testing compared to patients without VRE (35% vs 21%, p < 0.01). Of 761 patients who underwent subsequent stool PCR testing, 131 (17%) were positive for VRE (Table 1). Patients colonized with VRE were less likely to be Hispanic (10.7% vs 18%, p = 0.03) and more likely to be exposed to any antibiotic after VRE screening (100% vs 77%, p < 0.01). Such patients had a longer median hospital LOS and ICU LOS, and a higher in-hospital mortality. There were no other statistically significant demographic or clinical differences between patients with and without VRE colonization.

**Table 1:** Baseline characteristics of patients with and without vancomycin resistant *Enterococcus* colonization who underwent testing with a gastrointestinal pathogen PCR panel or *Clostridium difficile* test

Variable	Total (n = 761)	No VRE (n = 630, 82.8%)	VRE (n = 131, 17.2%)	p value
Sex				
Male	387 (51%)	330 (52%)	57 (43.5%)	
Female	374 (49%)	300 (48%)	74 (56.5%)	0.07
Race				
Asian	31 (4.1%)	27 (4.3%)	4 (3.1%)	
Black	89 (11%)	77 (12%)	12 (9.2%)	
White	286 (38%)	231 (37%)	55 (42.0%)	
Other/unknown	355 (47%)	295 (47%)	60 (45.8%)	0.55
Ethnicity				
Hispanic	128 (17%)	114 (18%)	14 (10.7%)	
Non-hispanic	280 (37%)	220 (35%)	60 (45.8%)	
Unknown	353 (46%)	296 (47%)	57 (43.5%)	*0.03*
Age group				
18–29	58 (7.6%)	45 (7.1%)	13 (9.9%)	
30–49	137 (18%)	115 (18%)	22 (16.8%)	
50–69	366 (48%)	304 (48%)	62 (47.3%)	
> 70	200 (26%)	166 (26%)	34 (26.0%)	0.74
Residential zip code				
New York city	414 (54%)	342 (54%)	72 (55.0%)	
Tristate area	328 (43%)	273 (43%)	55 (42.0%)	
Other	19 (2.5%)	15 (2.4%)	4 (3.1%)	0.88
Laboratory values at VRE testing (median, IQR)				
Creatinine (mg/dL)	1.1 (0.8–2.1)	1.1 (0.7–1.1)	1.03 (0.8–2.0)	0.58
Albumin (g/dL)	2.8 (2.4–3.2)	2.8 (2.4–3.2)	2.7 (2.3–3.1)	0.11
White blood cell count (cells × 10^3^/µL)	10 (6.5–14)	10 (6.5–14)	9.6 (6.3–14)	0.74
Hematocrit (%)	33 (32–34)	33 (32–34)	33 (31–34)	0.19
Platelets (× 10^3^/µL)	138 (135–141)	138 (135–141)	137 (135–141)	0.11
Surgery after VRE test	246 (32%)	209 (33%)	37 (28%)	0.27
Mechanical ventilation after VRE test	450 (59%)	371 (59%)	79 (60%)	0.76
Immunosuppression exposure after VRE test	430 (57%)	348 (55%)	82 (63%)	0.12
Antibiotic exposure after VRE test				
Any antibiotic	719 (95%)	483 (77%)	131 (100%)	*< 0.01*
Vancomycin IV	571 (75%)	467 (74%)	104 (79%)	0.21
VRE-targeted therapy	15 (2.6%)	13 (2.7%)	2 (1.9%)	0.64
LOS (days; median, IQR)				
Hospital LOS	15 (8–31)	14 (7–29)	19 (10–44)	*< 0.01*
Intensive care unit LOS	4 (2–8)	4 (2–8)	5 (3–9)	*0.01*
In-hospital mortality	52 (6.8%)	36 (5.7%)	16 (12%)	*< 0.01*

**Fig. 1:**
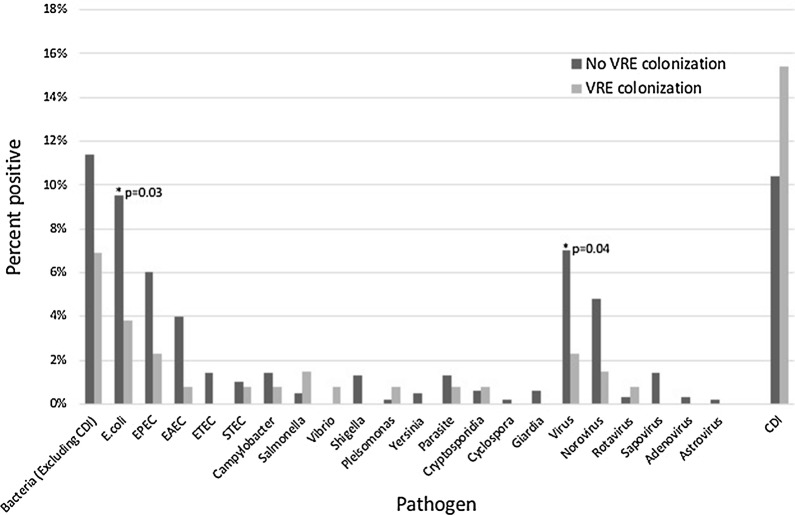
Percent of total tests positive for each pathogen or class of pathogens (* = significant; CDI = *Clostridium difficile* infection)

Over 5 years of data collection, patients colonized with VRE were less likely to subsequently test positive on the GI PCR compared to patients without VRE colonization (9.2% vs 18%, p = 0.01; Table 2) during an episode of diarrhea. Specifically, patients colonized with VRE had a lower proportion of GI PCR tests positive for *E. coli* species (3.8% vs 9.5%, p = 0.03) or viral (2.3% vs 7.0%, p = 0.04) enteric infections (Fig. 1). Of patients who underwent VRE screening, a subset of 716 were subsequently tested for *C. difficile*. Among these patients, 130 (18%) were positive for *C. difficile*. Patients colonized with VRE were more likely to test positive for *C. difficile* although this was not statistically significant (15% vs 10%, p = 0.11; Table 2, Fig. 1).

**Table 2:** Gastrointestinal pathogen PCR panel and *Clostridium difficile* test outcomes in patients with and without vancomycin resistant *Enterococcus* colonization

Variable	Total (n = 761)	No VRE (n = 630)	VRE (n = 131)	p value
GI PCR tests	761	630 (82.8%)	131 (17.2%)	
Positive	126 (17%)	114 (18.1%)	12 (9.2%)	*0.01*
Tests with more than one pathogen	34 (4.5%)	32 (5.1%)	2 (1.5%)	0.07
Place of test				
Emergency department	98 (12.9%)	81 (12.9%)	17 (13%)	
Endoscopy	7 (0.9%)	7 (1.1%)	0	
Inpatient	452 (60%)	373 (59.2%)	79 (60%)	
Outpatient	204 (27%)	169 (26.8%)	35 (27%)	0.69
Days from VRE test to GI PCR (median, IQR)	42 (5–463)	37 (5–495)	51 (6–479)	0.25
Test obtained during initial hospital stay	369 (48%)	312 (49.5%)	57 (43.5%)	0.21
*C. difficile* PCR tests	716	586 (81.8%)	130 (18.2%)	
Positive	81 (11%)	61 (10.4%)	20 (15%)	0.11
Place of test				
Inpatient	635 (100%)	586 (100%)	130 (100%)	–
Days from VRE test to CDI testing (median, IQR)	23 (4–370)	18 (4–351)	41 (5–503)	0.13
Test obtained during initial hospital stay	439 (61%)	364 (62%)	75 (58%)	0.11

**Fig. 2:**
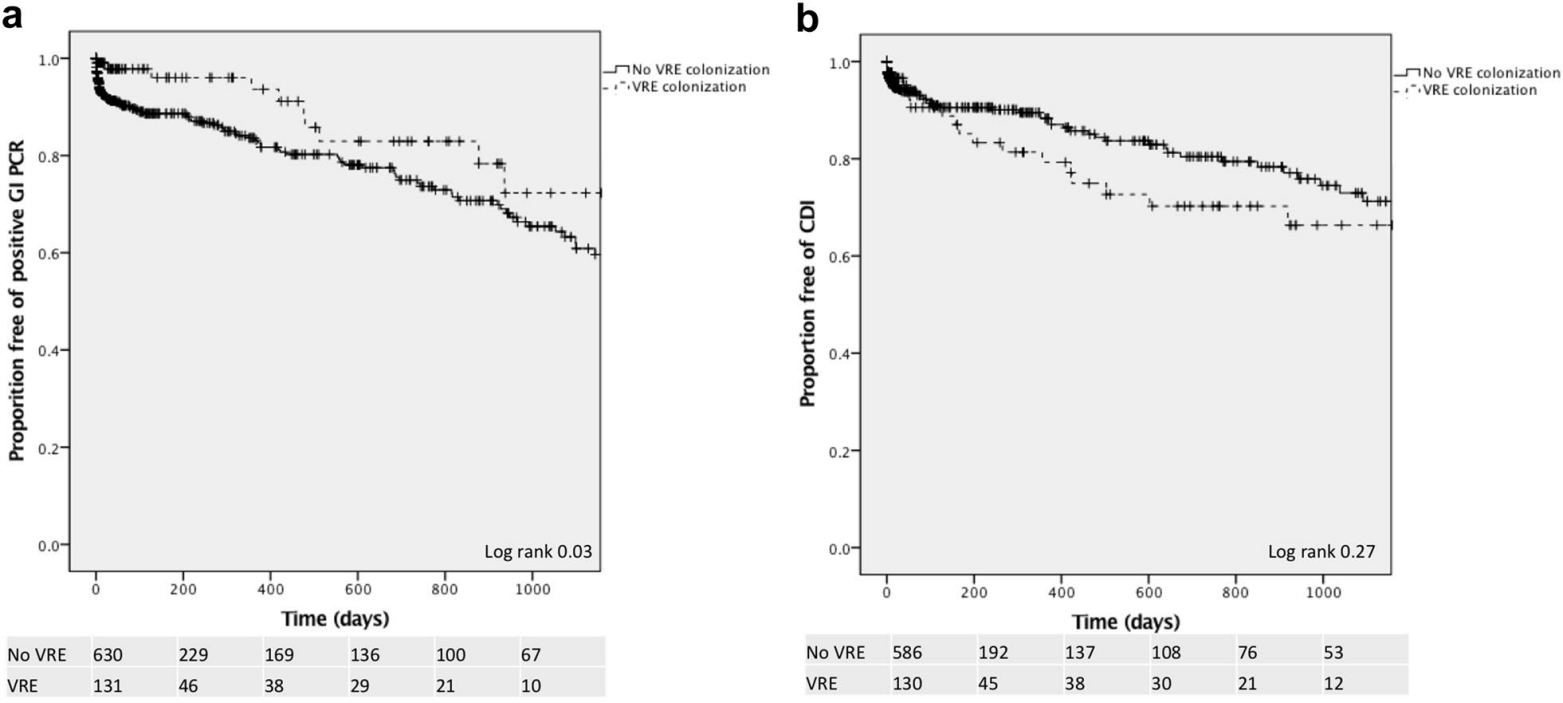
Time to positive gastrointestinal pathogen PCR panel (**a**) or positive *Clostridium difficile* PCR (**b**) stratified by VRE colonization status

On multivariable Cox regression analysis, patients with VRE colonization had a decreased risk of a positive GI PCR (aHR 0.47, 95% CI 0.25–0.88, p = 0.02, Table 3). This effect persisted over 5 years of follow up (log-rank 0.03, Fig. 2a). On excluding 325 patients who underwent stool testing within 30 days of VRE screening, on logistic regression, there was no change in this result (aOR 0.48, 95% CI 0.23–1.19, p = 0.04). Patients with a longer ICU LOS had an increased risk of *C. difficile* (aHR 1.02, 95% CI 1.01–1.04, p = 0.01; Table 3). There was a trend toward an increased risk of *C. difficile* in patients with VRE colonization (aHR 1.32, 95% CI 0.79–2.19, p = 0.21; log-rank 0.29, Fig. 2b).

**Table 3:** Predictors of a positive gastrointestinal pathogen PCR panel or *Clostridium difficile* test

Variable	GI PCR		*C. difficile* PCR	
	Univariable HR	Multivariable HR	Univariable HR	Multivariable HR
Sex				
Male	0.71 (0.49–1.02)		0.89 (0.60–1.31)	
Race				
Asian	0.99 (0.40–2.48)		0.48 (0.12–2.00)	
Black	0.68 (0.36–1.31)		0.66 (0.32–1.34)	
White	0.96 (0.65–1.40)		0.87 (0.57–1.32)	
Other/unknown	Reference		Reference	
Ethnicity				
Hispanic	1.23 (0.77–1.98)		1.16 (0.84–1.60)	
Non-hispanic	0.91 (0.61–1.36)		0.89 (0.68–1.17)	
Unknown	Reference		Reference	
Age group				
18–29	0.54 (0.21–1.38)		0.81 (0.34–1.95)	
30–49	1.17 (0.69–2.01)		0.75 (0.39–1.44)	
50–69	0.99 (0.64–1.53)		0.96 (0.61–1.50)	
> 70	Reference		Reference	
Residential zip code				
New York city	0.40 (0.15–1.12)		1.87 (0.26–13.51)	
Tristate area	0.42 (0.15–1.16)		1.84 (0.25–13.40)	
Other	Reference		Reference	
Laboratory values at VRE testing				
Creatinine (mg/dL)	0.89 (0.79–0.99)	0.89 (0.79–1.01)	0.98 (0.88–1.08)	
Albumin (g/dL)	1.08 (0.77–1.54)		0.82 (0.58–1.17)	
White blood cell count (cell/mcL)	1.01 (0.99–1.04)		1.02 (0.99–1.05)	
Hematocrit (%)	1.00 (0.97–1.03)		0.97 (0.95–1.00)	
Platelets (/mcL)	1.04 (1.01–1.08)	1.01 (0.97–1.05)	1.01 (0.97–1.06)	
VRE colonization	0.52 (0.28–0.96)	0.47 (0.25–0.88)	1.39 (0.83–2.27)	1.32 (0.79–2.19)
Surgery after VRE test	1.02 (0.70–1.49)		0.96 (0.63–1.48)	
Mechanical ventilation after VRE test	1.18 (0.82–1.70)		1.31 (0.88–1.95)	
Immunosuppression exposure after VRE test	1.00 (0.70–1.44)		1.10 (0.74–1.63)	
Antibiotic exposure after VRE testing				
Any antibiotic	0.93 (0.59–1.49)		1.20 (0.74–1.93)	
VRE–targeted therapy	0.72 (0.23–2.26)		1.02 (0.25–4.18)	
LOS				
Hospital LOS	1.01 (1.00–1.01)		1.00 (0.99–1.01)	
ICU LOS	1.01 (1.00–1.03)		1.02 (1.01–1.04)	1.02 (1.01–1.04)
In-hospital mortality	0.93 (0.41–2.13)		1.37 (0.50–3.72)	
Place of GI PCR test				
Emergency department	0.85 (0.45–1.61)			
Endoscopy	1.56 (0.37–6.60)			
Inpatient	0.87 (0.58–1.30)			
Outpatient	Reference

In examining the distribution of pathogens detected among those with positive stool PCR tests, 216 total pathogens were detected in patients without VRE colonization and 35 total pathogens were detected in patients with VRE colonization (Table 4). Of positive tests, there was a greater proportion of *C. difficile* among patients with VRE (57% vs 28%, p < 0.01) and a non-significant trend toward more bacterial infections (86% vs 76%, p = 0.20) and fewer viral infections (8.6% vs 20%, p = 0.11).

**Table 4:** Distribution of pathogens among those patients with a positive stool PCR result

	No VRE colonization	VRE colonization	p value
Total pathogens	216	35	
Bacteria	163 (76%)	30 (86%)	0.20
*Escherichia coli* (*E. coli*) species	60 (28%)	5 (14%)	0.10
Enteropathogenic *E. coli* (EPEC)	38 (18%)	3 (8.6%)	0.18
Enteroaggregative *E. coli* (EAEC)	25 (12%)	1 (2.9%)	0.14
Entertoxigenic *E. coli* (ETEC)	9 (4.2%)	0	0.37
Shiga toxin-producing *E. coli* (STEC)	6 (2.8%)	1 (2.9%)	0.66
Shigella/enteroinvasive *E. coli* (EIEC)	8 (3.7%)	0	0.38
*E. coli* O157	0	0	–
*Campylobacter* species	9 (4.2%)	1 (2.9%)	0.58
Salmonella	3 (1.4%)	2 (5.7%)	0.14
Vibrio species	0	1 (2.9%)	0.14
*Pleisomonas shigelloides*	1 (0.5%)	1 (2.9%)	0.26
*Yersinia enterocolitica*	3 (1.4%)	0	0.64
*Clostridium difficile*	61 (28%)	20 (57%)	< 0.01
Parasites	9 (4.2%)	1 (2.9%)	0.58
*Cryptosporidium*	4 (1.9%)	1 (2.9%)	0.53
*Cyclospora cayetanensis*	1 (0.5%)	0	0.86
*Entamoeba histolytica*	0	0	–
*Giardia lamblia*	4 (1.9%)	0	0.55
Virus	44 (20%)	3 (8.6%)	0.11
Norovirus (genogroups GI, GII)	30 (14%)	2 (5.7%)	0.27
Rotavirus A	2 (0.9%)	1 (2.9%)	0.36
Sapovirus (serotypes I, II, IV, V)	9 (4.2%)	0	0.37
Adenovirus F40/41	2 (0.9%)	0	0.74
Astrovirus	1 (0.5%)	0	0.86

## Discussion

In the present study, gut VRE colonization status predicted the risk of subsequent enteric infection as detected by broad, multiplex PCR stool testing. VRE colonization decreased the risk of a subsequent non-*C. difficile* enteric infection, a finding that persisted over 5 years of follow up. Although VRE colonization did not clearly impact subsequent *C. difficile*, it did modify the distribution of enteric infections such that *C. difficile* was the most common pathogen detected in VRE colonized patients with positive stool testing. These data have significant clinical implications in assessing the risk and management of enteric infection in patients colonized with VRE.

Recently, we have examined utilization of the GI PCR test in the general population and in specific populations, such as those with celiac disease and inflammatory bowel disease (IBD) [17–19]. This is the first analysis to focus on VRE and enteric infections utilizing multiplex stool PCR testing. Gut VRE colonization has a dramatic effect on the intestinal microbiome which may in turn protect against infection with non-*C. difficile* enteric pathogens. As such, the utility of broad stool PCR testing in patients with gut VRE colonization may be limited, and perhaps, should be restricted to *C. difficile* testing alone.

Physiologically, *enterococci* clones exhibit antibiotic resistance and starvation tolerance, which help them to thrive under hostile conditions including selective pressure from vancomycin [20]. In addition, selective eradication of Gram-negative bacteria by antibiotics reduces RegIII-γ levels and allows for the expansion of Gram-positive bacteria including *Enterococcus* [21]. Consequently, under certain conditions, *Enterococcus* exhibits a densely dominating phenotype in the gut [2]. In the setting of other ecological conditions, a more diverse set of pathogens may appear including multiple Gram negatives [22, 23]. These findings may explain why *Enterococcus,* while not a particularly virulent gut pathogen in isolation, is associated with all-cause infection and mortality [2, 9, 20]. VRE may dominate the gut microbiome, crowding out both healthy commensals and non-*C. difficile* enteric bacteria and viruses. Moreover, SagA, a secreted peptidoglycan hydrolase from *E. faecium* has demonstrated in vivo to trigger a protective intestinal epithelial cell program that limits the pathogenesis of enteric infections [1, 2]. In the present study, the protective effect of VRE persisted over time and is consistent with previous literature demonstrating low rates of VRE eradication, even after ICU and hospital discharge, and withdrawal of known risk factors [6–8].

*Clostridium difficile* is unique in that the relative abundance of the pathogen in the gut does not directly predict *C. difficile* carriage versus overt infection [24]. As a virulent, spore-forming and toxin-producing pathogen, gut microbiome abundance is not necessarily required to produce clinically significant symptoms [24, 25]. Among positive tests in patients with VRE colonization, *C. difficile* was the most common pathogen, consistent with the concept of VRE as a marker for a loss of gastrointestinal colonization resistance. Non-*C. difficile* enteric infections as detected by the GI PCR may not necessarily depend on loss of gastrointestinal colonization resistance but rather intrinsic pathogenicity and abundance under specific gut microbiome conditions. Several studies have suggested a possible link between *Enterococcus* and *C. difficile*, and our data may also suggest that VRE colonization and *C. difficile* share common gut ecological preferences, although this hypothesis requires further study [24, 26].

There are several limitations to this study. Patients colonized with VRE may receive more intense or longer duration courses of antibiotics, and it may be the antibiotics rather than VRE itself that confers and increased risk for *C. difficile* infection and a lower risk of other bacterial enteric infection. Although this study adjusted for antibiotics and nearly all patients were exposed, we had limited ability to adjust for differences in antibiotic duration or anti-anaerobic potency. Previous studies suggest that patients with VRE colonization have higher acute severity of illness at the time of ICU admission compared to patients who are not colonized. Such patients are also at increased risk for *C. difficile* and therefore, residual confounding, if present, would likely lead to an underestimate of the true protective effect of VRE colonization on enteric infections. While all patients screened for VRE were admitted to an ICU, there may be selection bias in patients who underwent stool PCR tests for diarrhea as patients with VRE were more likely to undergo stool testing. However, as stool testing generally leads to further findings, we believe these data likely underestimate the effect of VRE colonization on subsequent enteric infection. Moreover, although VRE colonization may persist for years, VRE status was not confirmed at same time as GI PCR or *C. difficile* PCR testing. In addition, PCR testing fails to discriminate between active infection and asymptomatic colonization, and there is considerable uncertainty regarding clinical interpretation and cost-effectiveness of such multiplex assays [27]. The GI PCR panel does not assess for the presence of Cytomegalovirus (CMV), a pathogen of increasing importance. Lastly, our sample size gave us limited ability to specify differences between enteropathogen types on the GI PCR panel.

In summary, VRE colonization was associated with a reduction in the risk of subsequent non-*C. difficile* enteric infection. Although changes in the gut microbiome likely underlie these findings, further study is required to specifically assess for the impact of VRE colonization on the gut microbiome and how these changes may directly impact acquisition of enteric pathogens.

## Methods

### Study population

We performed a retrospective cohort study using the electronic medical records of patients at New York Presbyterian-Columbia University Medical Center, a quaternary care institution in New York City. We identified all adult patients (≥ 18 years) admitted to any 1 of 9 distinct ICUs within our hospital network comprised of 2 large hospitals between 2012 and 2017 who were screened for VRE colonization via rectal swab and culture, and subsequently stool testing for diarrhea using a multiplex PCR assay. Patients were excluded if they had an ICU LOS less than 2 days or if they were diagnosed with an enteric infection by a GI PCR test before their VRE screen. GI or *C. difficile* PCR stool testing for diarrhea may have occurred during the index hospitalization or at any time point after discharge.

### Vancomycin-resistant *Enterococcus* screening

The primary exposure was VRE colonization, determined by the result of routine surveillance swabs for VRE which are performed on every patient within 1 h of ICU admission in our hospital network. Flocked rectal swabs were gathered by nurses with the patient in the left lateral decubitus position with the swab inserted deeply into the rectal canal and rotated 5 times. Swabs were transported in 1 mL of liquid Amies media for direct culture onto chromogenic differential media impregnated with 6 µg/mL of vancomycin (Remel). Plates were incubated at 33–37 ºC under aerobic conditions for 24 h and interpreted categorically according to the manufacturer’s instructions.

### Gastrointestinal pathogen PCR panel stool test

Gastrointestinal pathogens were identified using the FilmArray gastrointestinal pathogen PCR panel (GI PCR; BioFire Diagnostics, Salt Lake City, UT). This assay tests for 22 analytes in spontaneously voided stool including 13 bacteria, 5 viruses, and 4 parasites including *Campylobacter* (*jejuni, coli,* and *upsaliensis*), *Plesiomonas shigelloides*,* Salmonella*, *Yersinia enterocolitica*, *Vibrio (parahaemolyticus, vulnificus*, and *cholerae*), enteroaggregative *Escherichia coli* (*E. coli;* EAEC), enteropathogenic *E. coli* (EPEC), enterotoxigenic *E. coli* (ETEC), Shiga-like toxin-producing *E. coli* (STEC), *E. coli* O157, Shigella/enteroinvasive *E. coli* (EIEC), *Cryptosporidium* spp., *Cyclospora cayetanensis*, *Entamoeba histolytica, Giardia lamblia*, adenovirus (AdV) F40/41, astrovirus, norovirus GI/GII, rotavirus A, and sapovirus (I, II, IV, and V). The GI PCR is capable of the simultaneous detection and identification of nucleic acids from multiple bacteria, viruses, and parasites directly from stool samples in Cary Blair transport media. The multiplex PCR process takes approximately 1 h. The clinical sensitivity and specificity is 94.5–100% for all targets [14, 28].

### *Clostridium difficile* testing

*Clostridium difficile* PCR testing was done via PCR for the toxin B gene (Xpert *C. difficile*, Cepheid, Sunnyvale, CA). While the GI PCR includes testing for *C. difficile* toxins A and B at some institutions, this is not the case at our institution. *C. difficile* testing needed to be ordered separately, which was done at the discretion of providers. Therefore, some patients in this cohort did not receive testing for *C. difficile*. All patients, however, underwent testing with a GI PCR test.

### Co-variables

Automated queries were used to extract the following values from the medical record: dates of PCR tests, PCR results, date of birth, zip code, place of GI PCR test (e.g. emergency department, outpatient visit, inpatient hospitalization, endoscopy), sex, race, ethnicity, laboratory values at the time of VRE screening, history of surgery, mechanical ventilation, immunosuppression exposure, antibiotic exposure, hospital and ICU LOS after VRE screening. Exposure to linezolid or daptomycin was subclassified as VRE-directed antimicrobials.

### Outcomes and statistical analyses

Chi square tests were used to compare categorical variables and the *t* test or Mann–Whitney test for continuous variables. Linearity of the continuous variables was assessed visually by plotting residuals against predicted values and examining for a bowed pattern. When cell counts were less than 5, Fisher’s exact test was used. To account for time from VRE screening to enteric infection testing, we evaluated outcomes using a Cox proportional hazards model, with observation time beginning at the time of VRE screening and ending upon a positive GI PCR test, patient death, or their last clinical encounter at our medical center. Survival curves were constructed to illustrate differences in time to outcome. To build the final model, a full model was constructed including all possible predictors of enteric infections, and co-variables were removed in a stepwise manner if they had no independent relationship with the outcome (p > 0.05) or if they did not alter the relationship between VRE colonization status and GI PCR result (< 10% change in the beta-coefficient representing VRE colonization), and adjusted hazard ratios (aHR) were reported. Logistic regression was also performed to assess predictors of enteric infection to confirm our above analyses. We then constructed a logistic regression model limited to GI PCR tests obtained within 30 days of a VRE test and report an adjusted odds ratio (aOR). All tests were considered significant at a 2-sided p value less than 0.05. SPSS software (IBM) was used to perform all statistical analyses.
